# Geography, Antimicrobial Resistance, and Genomics of *Salmonella enterica* (Serotypes Newport and Anatum) from Meat in Mexico (2021–2023)

**DOI:** 10.3390/microorganisms12122485

**Published:** 2024-12-03

**Authors:** Eduardo Canek Reynoso, Enrique Jesús Delgado-Suárez, Cindy Fabiola Hernández-Pérez, Yaselda Chavarin-Pineda, Elizabeth Ernestina Godoy-Lozano, Geny Fierros-Zárate, Omar Alejandro Aguilar-Vera, Santiago Castillo-Ramírez, Luz del Carmen Sierra Gómez-Pedroso, Luisa María Sánchez-Zamorano

**Affiliations:** 1 Centro de Investigación en Salud Poblacional, Instituto Nacional de Salud Pública (INSP), Morelos 62100, Mexico; eduardo.reynoso@insp.edu.mx (E.C.R.); gsfierro@insp.mx (G.F.-Z.); 2 Facultad de Medicina Veterinaria y Zootecnia, Universidad Nacional Autónoma de México (UNAM), Ciudad de México 04510, Mexico; ejds@fmvz.unam.mx (E.J.D.-S.); luzsierra@fmvz.unam.mx (L.d.C.S.G.-P.); 3 Centro Nacional de Referencia de Inocuidad y Bioseguridad Agroalimentaria, Servicio Nacional de Sanidad, Inocuidad y Calidad Agroalimentaria (SENASICA), Tecámac 55740, Mexico; dgiaap.iica44@senasica.gob.mx; 4 Centro de Investigación en Ciencias Agrícolas, Instituto de Ciencias, Benemérita Universidad Autónoma de Puebla (BUAP), Puebla 72570, Mexico; 5 Centro de Investigación Sobre Enfermedades Infecciosas, Instituto Nacional de Salud Pública (INSP), Morelos 62100, Mexico; elizabeth.godoy@insp.mx; 6 Centro de Ciencias Genómicas, Universidad Nacional Autónoma de México (UNAM), Morelos 62210, Mexico; aaguilar@ccg.unam.mx (O.A.A.-V.); iago@ccg.unam.mx (S.C.-R.)

**Keywords:** antimicrobial resistance, *Salmonella enterica*, Newport, Anatum, pangenome

## Abstract

*Salmonella enterica* non-typhoidal is a major contributor to diarrheal diseases, with over 2600 serovars identified across diverse environments. In Mexico, serovars Newport and Anatum have shown a marked increase, especially in foodborne disease, posing a public health problem. We conducted a cross-sectional study from 2021 to 2023 using active epidemiological surveillance to assess contamination in ground beef and pork at butcher shops nationwide. It involved isolation, phenotypic antimicrobial resistance, comparative genomics, spatial distribution, antimicrobial-resistance genes, and pangenome analysis. A total of 402 non-typhoidal *S. enterica* strains were isolated, including 59 Newport and 50 Anatum. After curating for redundancy, 45 Newport and 32 Anatum strains remained. We found that 75% of Newport strains exhibited multidrug resistance (MDR), compared to 25% of Anatum strains. *Salmonella* Newport also showed a broader distribution and stronger antibiotic-resistance capacity, particularly due to genes such as *mphA* and *ramA*. Our pangenome analysis showed a predominance of cell maintenance and survival-process genes in the accessory genome of both serotypes. Considering unique genes, *Salmonella* Anatum and Newport showed a notorious abundance of genes with functions related to replication, recombination, and repair. The substantial rise of Anatum and Newport strains in meat samples for human consumption presents an epidemiological alert, highlighting the critical need for stringent surveillance programs to mitigate human and ecosystem health risks.

## 1. Introduction

*Salmonella enterica* is a Gram-negative bacillus capable of surviving in diverse environmental conditions and pressures. Its high invasiveness and infectivity make it a significant concern for public health, veterinary health, food safety, and ecosystem stability [1,2]. As one of the four leading causes of diarrheal disease worldwide [3], *S. enterica* infects approximately 200 million people annually, with an estimated mortality rate of 0.08% [4,5,6]. In Mexico, 66,138 new cases of *S. enterica* were reported in 2023, with 21.6% linked to typhoid fever, 9.7% to paratyphoid, and 68.7% to another salmonellosis [7].

*S. enterica* includes over 2600 serotypes across five subspecies: *S. enterica*, *S. salamae*, *S. arizonae*, *S. diarizonae*, *S. houtenae*, and *S. indica*. The main subspecies that exhibits pathogenicity in humans and animal-based foods is *S. enterica*, which includes more than 1500 serotypes and is responsible for gastrointestinal infectious diseases [8,9]. According to its human pathology, *S. enterica* is divided into typhoidal and non-typhoidal types [10]. Non-typhoid salmonellosis represents a significant global public health problem due to its phylogenetic diversity, given its genomic heterogeneity, antimicrobial resistance, and survival in environmental conditions [11]. The geographic distribution and host range of serotypes vary by region and are influenced by the genomic characteristics presented [12]. The serotypes Newport and Anatum are important sources of salmonellosis in animals and have significantly increased resistance to multiple antibacterial agents, largely due to the horizontal transfer of resistance genes from other animal and agricultural-associated microorganisms [13,14,15].

Anatum is one of the most prevalent serotypes globally and in Mexico, showing notable persistence in cattle [16]. Outbreaks have been found in the USA, with strong antimicrobial-resistance profiles in travelers and seafood from Taiwan [17]. Although no genetic relationship with these strains has been found in Mexico, the potential of this serotype to adapt to different environmental pressures and spread rapidly has been determined. For instance, this serotype has become a public health problem in Asian countries [18,19]. Notably, it ranks among the top five most prevalent serotypes in Latin America. Although the presence in México of *Salmonella* Anatum in pork meat has been determined to be rare, it is the most pervasive in beef in this region of the world and North America, making it “the most noteworthy serotype in beef” [15,20,21,22]. However, studies on its pathogenicity remain limited.

In 2016, the Centers for Disease Control and Prevention (CDC) identified the presence of *Salmonella* Newport multidrug-resistant (MDR) across the US in all 50 states, prompting a monitoring program. This program identified a sustained rise in Newport cases from 2021 to 2022, with rates nearly double those observed from 2018 to 2020, particularly in travelers to Mexico who had consumed beef or Mexican cheese [23]. In Mexico, *Salmonella* serotype Newport is among the most prevalent serotypes in animal-derived foods [21,24], fish [25], water [26], and fruits and vegetables [27]. Furthermore, this serotype has shown a marked increase in resistance profiles [24].

Given the rise in Anatum strains in beef and pork and the notable antimicrobial resistance in the Newport serotype, mainly to macrolides, this study presents a surveillance effort to monitor these two serotypes of *Salmonella enterica* in beef and pork from butcher shops across 28 cities of the Mexican Republic from 2021 to 2023.

## 2. Materials and Methods

### 2.1. Sample Collection and Isolates

A total of 681 ground meat samples, comprising 287 pork (*Sus scrofa*) and 389 beef (*Bos taurus*), were collected using convenience sampling from 193 retail outlets. Our sampling included direct-to-public sellers and stores in the main cities or capitals of 28 out of 32 states in Mexico, and the sampling period spanned from May 2021 to June 2023. The cities were selected for their economic significance and accessibility to air transportation. To ensure sample viability, most were transported by air. Supplementary Table S1 provides details on the cities and states sampled. This study did not involve live animals and was reviewed and approved by the National Institute of Public Health Research Ethics Committee (ID 1716), Biosafety Committee (ID 1707), and Research Committee (ID 1716).

Our previous studies [24,26,28] provide details of the isolating and identifying Salmonella procedure. In brief, 25 g of meat sample was blended in peptone water; spiked in Rappaport-Vassiliadis and tetrathionate broths; and then plated on selective media on Hektoen enteric, xylose–lysine–desoxycholate, and bismuth sulfite agar. Once characteristic *Salmonella* isolates were present, their identity was confirmed using biochemical tests (triple sugar iron agar, lysine iron agar, and urea tests) and PCR (amplification of a 500 bp fragment of the constitutive gene, *invA*) [24].

### 2.2. Geographic Information Systems

The maps were created using the QGIS 3.28 geographic information system. The vector layer of Mexico’s political divisions (CONABIO, 2024) was utilized for the species distribution map. A new field was added to the attribute table to include the sample type and classify it by meat type. Next, the layer representing the sampling sites was overlaid, with dots indicating the types of strains found (Anatum or Newport). Additionally, the ESRI Terrain base map was used. The phenotype map was constructed based on the number of resistance genes and was categorized by size.

### 2.3. Antibiotic Susceptibility Testing

The *Salmonella* strains found in beef and pork were tested for antibiotic susceptibility using a Kirby–Bauer disk diffusion protocol [29] with 12 critical antibiotics recommended by the World Health Organization [30]. The antibiotics tested were amikacin (AMK), amoxicillin/clavulanic acid (AMC), ampicillin (AMP), azithromycin (AZM), cefepime (FEP), ceftriaxone (CRO), chloramphenicol (CHL), ciprofloxacin (CIP), meropenem (MEM), streptomycin (STR), tetracycline (TET), and sulfamethoxazole/trimethoprim (SXT) [31]. *Escherichia coli* (ATCC 8739) and *Pseudomonas aeruginosa* (ATCC 27853) strains were used as controls. The susceptibility of the strains was evaluated based on the criteria provided by the Clinical and Laboratory Standards Institute (CLSI). A strain was considered pan-susceptible if it was susceptible to all antibiotics, resistant if it was resistant to one or two classes of antibiotics, multidrug-resistant (MDR) if it was resistant to three or more classes of antibiotics, extensively drug-resistant (XDR) if it was susceptible to one or two classes of antibiotics, and pan drug-resistant (PDR) when it was resistant to all antimicrobial agents to which it was exposed [32].

### 2.4. Extraction, Whole-Genome Sequencing (WGS), and Genome Assembly

DNA extraction and WGS processing were conducted at the National Reference Centre for Food Safety and Biosecurity. The genomic DNA (gDNA) was extracted using the DNeasy Blood and Tissue Kit (Qiagen, Hilden, Germany), following the supplier’s instructions. The purity of the DNA was assessed using a Qubit 3.0 fluorometer (Invitrogen, Carlsbad, CA, USA) and NanoDrop One (Thermo Fisher Scientific, Waltham, MA, USA) by measuring the absorbance at 260 and 280 nm. The extracts with a minimum concentration of 10 ng/μL and an optimal 260/280 ratio (~1.8) were used for further analysis.

Libraries were prepared and sequenced from 200 ng of gDNA using the Illumina DNA Prep Kit (Illumina, San Diego, CA, USA), following the manufacturer’s protocol. IDT for Illumina DNA/RNA UD indexes was used for the indexing step. Library quality was assessed using the Qubit 3.0 fluorometer and the 4200 TapeStation system (Agilent, Santa Clara, CA, USA). The normalized library was sequenced on the NextSeq 500 system (Illumina platform) using the NextSeq Reagent Kit V2.5 (300 cycles). SRA sequences are available at the NCBI site (https://www.ncbi.nlm.nih.gov/; accessed on 28 November 2024), under the BioProject Accession PRJNA480281. The quality of the raw sequences was checked using FastQC (v0.11.9; Babraham Bioinformatics; Cambridge, UK) [33]. Contaminations and sequences with poor Phred values (Q < 30) were removed with Trimmomatic (v0.39; Usadel Lab; Jülich, North Rhine-Westphalia, Germany) [34]. We ensured high-quality trimmed sequences by reanalyzing with FastQC. For de novo genome assembly, we utilized the Pathogen Resource and Integration Center (PATRIC) web server [35] with SPAdes version (v3.13.1; Center for Algorithmic Biotechnology, St. Petersburg State University; St. Petersburg, Russia) [36].

Assemblies were evaluated to have no more than 300 contigs, with a fragmentation index as low as possible (L50), depth of coverage ≥ 30×, % guanine–cytosine (GC) of 50 ≤ GC ≤ 53, and between 4.5 and 5.1 Mbp, which are typical values for *Salmonella* [37]; these parameters were evaluated with the QUAST (Quality Assessment Tool for Genome Assemblies; v5.2.0; Center for Algorithmic Biotechnology, St. Petersburg State University; St. Petersburg, Russia) [38].

### 2.5. Bioinformatics Analysis

The Newport and Anatum serotypes were identified using SeqSero2 (v1.2.2; Centers for Disease Control and Prevention; Atlanta, GA, USA) [39] and confirmed with *Salmonella* In Silico Typing Resource (SISTR; v1.1.2; Public Health Agency of Canada; Winnipeg, MB, Canada) [40]. Multi-locus sequence typing (MLST) based on sequences from seven housekeeping gene fragments from pubMLST (https://pubmlst.org/bigsdb?db=pubmlst_salmonella_seqdef; accessed on 28 November 2024) was used to predict the Sequence Type (ST) [41]. To compare genomes from the Newport and Anatum strains, we calculated the average nucleotide identity (ANI) using pyANI (v0.2.7; Python Software Foundation; Wilmington, DE, USA), with the align option, with MUMmer3 (ANIm) [42,43].

To identify antimicrobial-resistance genes (ARGs) and perform plasmid profiling with the assembled contigs and serotype prediction, we used ABRicate (v1.0.1; University of Oxford; Oxford, UK) (https://github.com/tseemann/abricate; accessed on 28 November 2024). For ARG identification, we utilized the Comprehensive Antibiotic Resistance Database (CARD; http://arpcard.mcmaster.ca; accessed on 28 November 2024) for the resistome prediction [44,45].

Whole genomes were annotated using Prokka (v1.14.5; University of Melbourne; Melbourne, Victoria, Australia) [46], with a locus tag counter increment of one, a minimum contig size of 200, and a similarity e-value cut-off of 0.000001. Following this, we performed a pan-genome analysis using Roary (v3.13.0; University of Edinburgh; Edinburgh, Scotland, UK) [47]. The analysis applied a minimum percentage identity of 95% when considering the BLASTp searches, allowing for a maximum of 50,000 clusters. The annotated genomes served as input for Roary, enabling us to determine the quantities of core and accessory genes, which include soft-core, shell, and cloud genes.

### 2.6. Statistical Analysis and Data Visualization

Descriptive statistical analyses were performed using R software (v4.2.3) with tidyverse (v2.0.0; RStudio; Boston, MA, USA) package [48]. The data were visualized through various graphical representations. UpSet plot originated through the ComplexUpset (v1.6.3; RStudio; Boston, MA, USA) package in R [49]. Heatmaps were generated using the ComplexHeatmap (v2.13.1; RStudio; Boston, MA, USA) package in R [50,51]. Histograms were created using ggplot2 (v3.5.1; RStudio; Boston, MA, USA) [52]. Additional visualizations were designed using Flouris (https://flourish.studio; accessed on 28 November 2024). The methodology described by Szklo and Nieto (2000) was used to determine the percentage of positive agreement [53].

## 3. Results

### 3.1. Geographic Distribution

**Figure 1:**
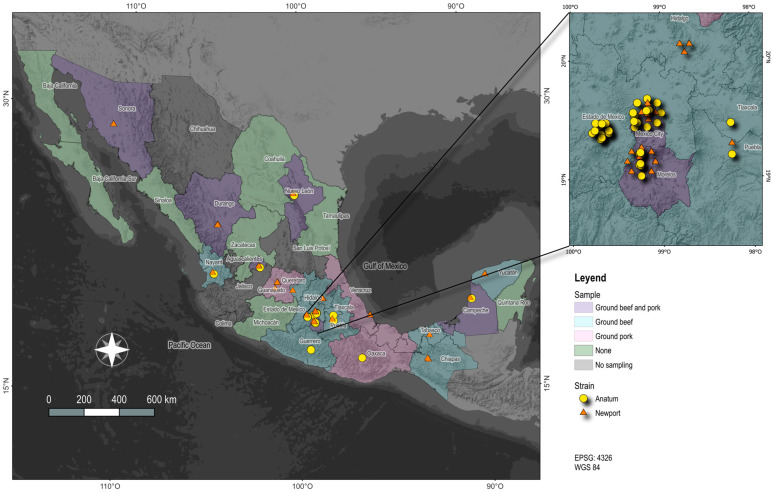
Spatial distribution in Mexico of *Salmonella enterica* serotype Newport and Anatum in ground beef and pork.

Between May 2021 and June 2023, 402 non-typhoidal *Salmonella enterica* strains were identified in the samples of ground beef (226 strains) and pork (172 strains) from 20 of 32 Mexican states. Figure 1 shows the distribution of *Salmonella enterica* serotype Newport (59 strains; 14.7%) and Anatum (50 strains; 12.4%).

In ground beef, 13.7% of the isolated strains belonged to the Newport serotype, while 16.4% were of the Anatum serotype. For ground pork, 16.3% were identified as Newport serotype, and 7.6% as Anatum serotype. Mexico City recorded the highest number of Newport and Anatum isolates (19% and 14.4%, respectively), followed by Toluca and Cuernavaca, each with 15 isolates (13.8%). Supplementary Figure S1 shows the total distribution of Newport and Anatum isolates in the different cities.

### 3.2. Comparative Analysis of Salmonella Newport and Anatum Genomes

After sequencing the complete genome of the 109 isolates from Newport and Anatum, 32 isolates were found and discarded because they corresponded to the same strain, according to 100% identity of the complete genome, and came from the same meat sample. Considering the Newport serotype, nine isolates were discarded from ground beef, three in Mexico City, three in Durango, two in Pachuca, and one in Tuxtla Gutiérrez; and in ground pork, five isolates were discarded, three in the Port of Veracruz, one in Mexico City, and one in Hermosillo. For Anatum, 13 isolates were found in ground beef, 6 in Toluca, 3 in Mexico City, 2 in Cuernavaca, 1 in Aguascalientes, and 1 in Chilpancingo; and 5 isolates were discarded in ground pork, 3 in Mexico City, and 2 in the City of Oaxaca.

**Figure 2:**
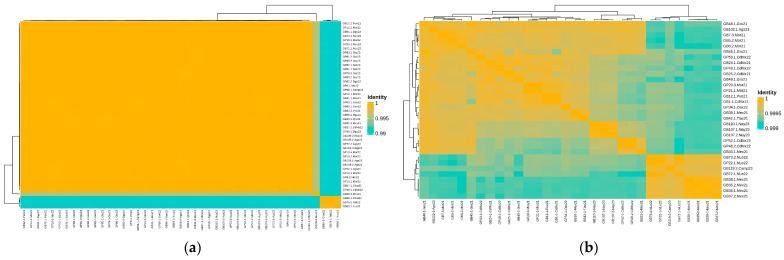
Average nucleotide identity of *Salmonella enterica* serotype Newport (**a**) and Anatum (**b**).

The ANI analysis of the 45 Newport isolates (Figure 2a) shows minimal variation in the genomes of most isolates. However, three samples from southern regions of the country (Tuxtla Gutierrez, Villahermosa, and Merida) obtained from ground beef show a 0.01 (1%) variation compared to the rest of the isolates for this serotype. The ANI analysis for the Anatum isolates shows even less variation among their genomes <0.001 (<0.1%) (Figure 2b). In addition, there is a noticeable correlation between the genomes and the cities in which the samples were collected, regardless of the animal origin of the sample. Taken together, these results show very little variation in both serotypes but even more so in Anatum.

### 3.3. Phenotypic and Genotypic of Antimicrobial-Resistance Profiles

**Figure 3:**
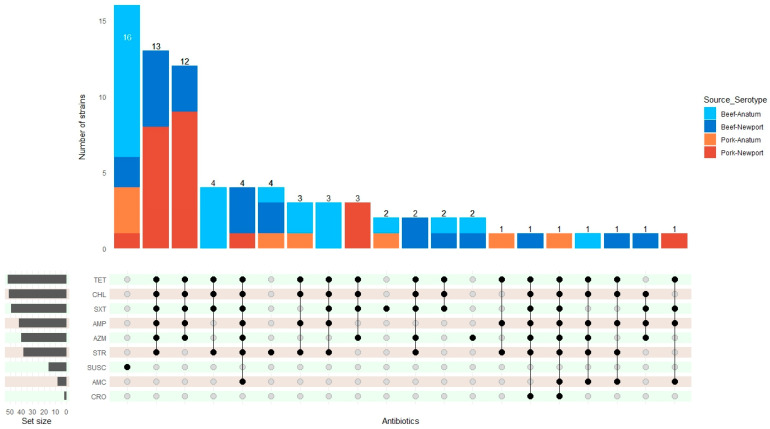
Relationship of Newport and Anatum serotypes and sample source to the number of phenotypically resistant strains. “Set size” represents the number of resistant strains per antibiotic. SUSC, strain susceptible to all antibiotics.

Out of 77 isolates representing both serotypes, 16 were susceptible to all tested antimicrobials (3 Newport and 13 Anatum). All isolates exhibited 100% susceptibility to AMK, FEP, ciprofloxacin CIP, and MEM. Eight strains showed resistance to only one antimicrobial. Two were resistant to AZM (one in each serotype), two to SXT (both in Anatum), and four to STR (two for each serotype). Figure 3 illustrates the distribution of MDR isolates: 39 for Newport and 14 for Anatum. TET and CHL had the highest number of resistant strains, 52 and 51, respectively. Among the resistant strains to CHL, 74.5% were from Newport and 25.5% from Anatum. For TET, the distribution was 72.5% in Newport and 27.5% in Anatum; all isolates resistant to these two antimicrobials were MDR. SXT was the third antimicrobial with higher resistant organisms, totaling 49 isolates (47 MDR), with 77.6% from Newport and 22.4% from Anatum. Ampicillin (AMP) had 41 resistant strains, all MDR, with 78% from Newport and 22% from Anatum. AZM showed 40 resistant isolates (38 MDR), 92.5% from the Newport serotype and 7.5% from Anatum. STR had 38 resistant isolates (34 MDR), comprising 60.5% from Newport and 39.5% from Anatum. Eight resistant isolates were identified for AMC, all classified as MDR, with 75% from Newport and 25% from Anatum. Finally, there was one resistant isolate each from Newport and Anatum for CRO, which were also MDR. The strongest correlations were observed with CHL/SXT and CHL/TET found in 49 of 53 MDR microorganisms. AMP/TET followed this with 46 strains, SXT/TET with 45, and AMP/CHL with 39 strains. Additionally, 38 strains resisted CHL/AZM, while AZM/SXT had 37 isolates. The relationships of 36 AZM/TET, AMP/AZM, and AMP/SXT were seen in 35 strains each. SRT/CHL and SRT/TET were associated with 33 strains, and SRT/SXT with 28 strains. For AMP/SRT, there were 27 resistant strains, and 21 strains exhibited resistance to AZM/SRT. The antimicrobial agents with the lowest incidence of resistance were AMC and CRO.

**Figure 4:**
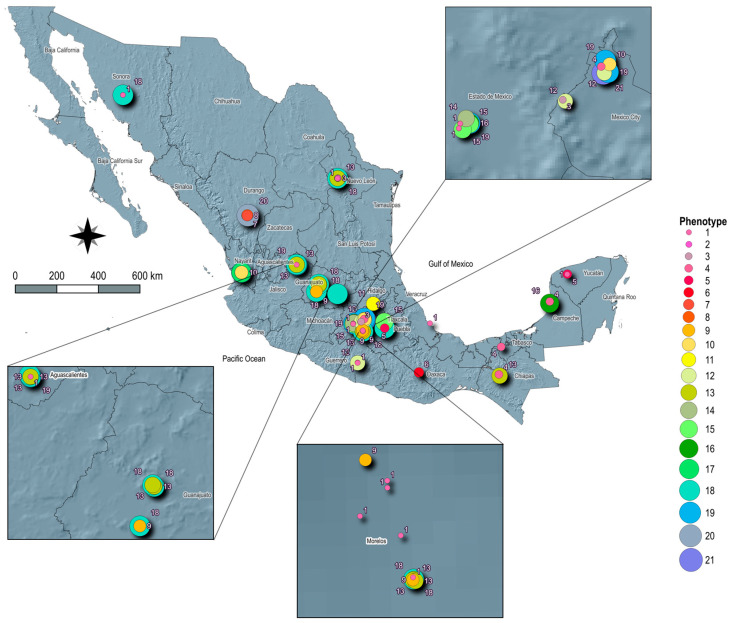
Spatial distribution of phenotypic resistance. The numbers represent the class of antimicrobial or combination and if the strain is pan-susceptible: 1, pan-susceptible; 2, AZM; 3, SXT; 4, SRT; 5, CHL/SXT/TET; 6, STR/AMP/TET; 7, STR/AMP/TET; 8, AMP/CHL/SXT/AZM; 9, AMP/CHL/SXT/AZM; 10, STR/AMP/CHL/TET; 11, STR/AMP/CHL/TET; 12, STR/CHL/SXT/TET; 13, STR/CHL/SXT/TET; 14, STR/AMP/AMC/CHL/TET; 15, STR/AMP/CHL/SXT/TET; 16, STR/CHL/SXT/TET/AZM; 17, STR/AMP/AMC/CHL/TET/AZM; 18, STR/AMP/CHL/SXT/TET/AZM; 19, STR/AMP/AMC/CHL/SXT/TET/AZM; 20, STR/AMP/CRO/CHL/SXT/TET/AZM; 21, STR/AMP/AMC/CRO/CHL/SXT/TET/AZM.

The distribution of phenotypic resistance is illustrated in Figure 4. One strain of *Salmonella* Anatum, collected from ground pork in Mexico City, was resistant to eight antimicrobials: STR, AMP, AMC, CRO, CHL, SXT, TET, and AZM. Additionally, five strains of *Salmonella* Newport were resistant to seven antimicrobials, with four strains showing resistance to STR, AMP, AMC, CHL, SXT, TET, and AZM. These strains were identified in Aguascalientes, Mexico City, and Toluca, with three detected in ground beef and one in ground pork. Another strain from the state of Durango (also found in ground beef) exhibited resistance to STR, AMP, CRO, CHL, SXT, TET, and AZM. A typical combination of phenotypic resistance (STR, AMP, CHL, SXT, TET, and AZM) was observed in 12 *Salmonella* Newport strains (5 in ground beef and 7 in ground pork) across eight cities in Mexico, including Aguascalientes, Tepic, Puebla, Queretaro, Cuernavaca, Monterrey, Hermosillo, and Guanajuato, primarily in the central and northern regions. Another frequent resistance combination—AMP, CHL, SXT, TET, and AZM—was also recorded for 12 *Salmonella* Newport strains (3 in ground beef and 9 in ground pork) in five cities (Aguascalientes, Tuxtla Gutiérrez, Monterrey, Cuernavaca, and Guanajuato), spanning central, southern, and northern Mexico. Other tested combinations of phenotypic antimicrobial resistance were found in fewer than three strains.

**Figure 5:**
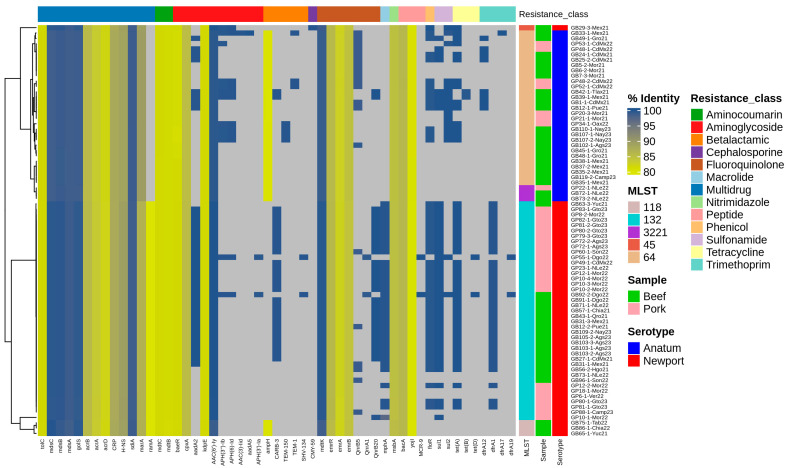
Profiles of antimicrobial-resistance genes found in *Salmonella enterica* serotype Newport and Anatum genomes in beef and pork. The gray color indicates no presence of the resistance gene.

The analysis of antimicrobial-resistance genes using the CARD revealed 12 clusters of different antimicrobial classes and an additional cluster associated with multidrug resistance. The aminoglycoside group exhibited the highest number of genes in the analysis, totaling ten. Four genes—*baeR*, *kdpE*, *cpxA*, and *aac(6′)-ly*—were present in 100% of the microorganisms, with percentage identities of 81%, 81%, 85%, and 99.5%, respectively. The *aadA2* gene was identified in 30 Newport isolates and 8 Anatum isolates, with 100% identity. The *aph(3″)-lb* and *aph(6)-ld* genes were present in three Newport strains, with *aph(3″)-lb* found in one additional Anatum strain (totaling ten) and *aph(6)-ld* in one more strain (totaling nine). The *aph(3′)-la* gene was present in only two Newport strains. The *aac(3)-lid* and *aadA5* genes were each found in only one Anatum strain (Figure 5).

The fluoroquinolone-resistance group contained four genes in 100% of the isolates: *mdtK*, *emrA*, *emrB*, and *emrR*, with identities of 99%, 86%, 83%, and 85%, respectively. The *qnrB20* gene was found in 28 Newport and 2 Anatum strains; the *qnrB5* gene was present in 6 Newport and 14 Anatum strains; and the qnrA1 gene was detected in only 2 Newport strains.

The *ampH* gene was found in 45% of the isolated microorganisms for beta-lactam compounds, specifically in 32 Anatum strains and 3 Newport strains. The CARB-3 gene was observed in 27 Newport and 2 Anatum strains. The TEM-1 gene and TEM-150 gene were found solely in the Anatum serotype, with three and four occurrences, respectively. In contrast, the SHV-134 gene was identified only in Newport, present in two strains.

In the trimethoprim-resistance group, two genes—*dfrA1* and *dfrA19*—were found exclusively in Newport, with 34 and 2 strains, respectively. Meanwhile, the *dfrA12* and *dfrA17* genes were present only in Anatum, with eight and one strain, each.

The tetracycline-resistance group had three genes, with *tetA* being the most prevalent, found in 35 Newport and 13 Anatum strains. The other two genes, *tetB* and *tetC*, were present in two strains each of Anatum and Newport, respectively. The antibiotic peptide-resistance genes, *bacA* and *yojI*, were present in all Newport and Anatum strains, while the MCR-9 gene appeared in two Newport strains.

Additionally, the *mdtB* and *mdtC* genes, conferring aminocoumarin resistance, were found in all isolates, and the sulfonamide-resistance genes, *sul1* and *sul2*, were detected in 44 (36 Newport and 8 Anatum) and 17 (3 Newport and 14 Anatum) strains, respectively.

Cephalosporins, macrolides, nitroimidazoles, and phenicols each exhibited one resistance gene. In the cephalosporin group, the CMY-59 gene was identified in one Newport strain. The *mphA* gene confers macrolide resistance was found in 35 Newport strains. For the nitroimidazole group, the *msbA* gene appeared in all tested microorganisms. Lastly, in the phenicol group, the *floR* gene was detected in 35 Newport strains and 13 Anatum strains.

As illustrated in Figure 5, all isolates contained genes associated with multidrug resistance, including *tolC*, *mdsC*, *mdsB*, *mdsA*, *golS*, *acrB*, *acrA*, *acrD*, CRP, H-NS, *sdiA*, and *marA*. However, the *ramA* gene was found in 44 out of the 45 Newport strains, while it was absent in the Anatum strain.

We identified five sequence types: three in Newport (ST132 at 91%, ST118 at 7%, and ST45 at 2%) and two in Anatum (ST64 at 91% and ST3221 at 9%).

**Figure 6:**
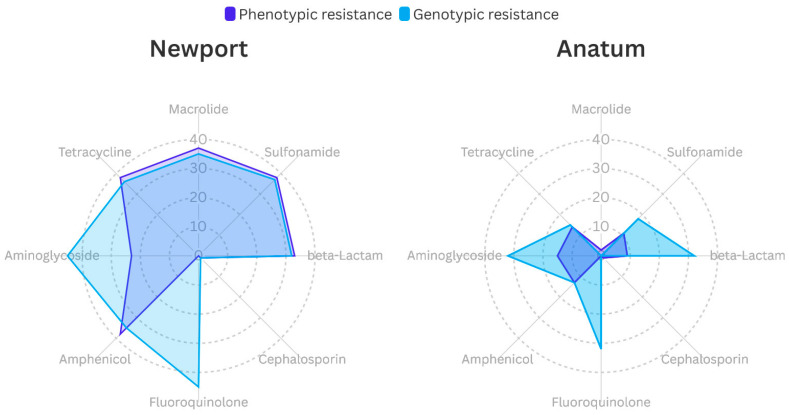
Phenotypic vs. genotypic resistance of *Salmonella enterica* serotype Newport and Anatum.

The phenotypic resistance of microorganisms was compared with the presence of resistance genes grouped into eight categories: aminoglycosides, amphenicols, fluoroquinolones, cephalosporins, beta-lactams, sulfonamides, macrolides, and tetracyclines. Figure 6 shows that *Salmonella* Newport has a significantly higher number of resistance genes than *Salmonella* Anatum and greater phenotypic resistance to all groups of antimicrobials. In the case of macrolides, the difference is notorious, as there were no resistance genes in Anatum. It is observed that Newport strains have a greater positive concordance (97.7%) between phenotypic resistance and antibiotic-resistance genes compared to Anatum strains (72.0%).

In both serotypes, a difference of 49% was observed for the aminoglycoside group, with phenotypic resistance present in 38 strains, while all microorganisms had at least one corresponding resistance gene. Resistance levels for amphenicols, beta-lactams, sulfonamides, and tetracyclines were similar in both evaluations, showing rates of 51%, 41%, 41%, 49%, and 51% for phenotypic resistance, compared to 48%, 38%, 55%, and 52% for genotypic resistance, respectively. In the macrolide group, phenotypic resistance was more pronounced, with 45 resistant strains identified, and the *mphA* gene was present in 40 strains of the Newport serotype. For cephalosporins, phenotypic resistance was observed in two microorganisms, with only one strain showing the CMY-59 gene, which confers resistance to this group. Finally, while all isolates in the fluoroquinolone group contained at least one resistance gene, all microorganisms were susceptible to ciprofloxacin.

### 3.4. Pangenome Analysis of Newport and Anatum Serotypes

Pangenome analysis reveals differences in the number and percentage of genes present across various *Salmonella enterica* strains, specifically Newport and Anatum serotypes. These genes can be categorized as CORE genes, essential in all strains; ACCESSORY genes, present in some but not all strains; and UNIQUE genes, found in only a single strain. The differences in these genes suggest potential adaptations between the two serotypes, particularly in functions related to replication, repair, transport, and the metabolism of different compounds.

**Figure 7:**
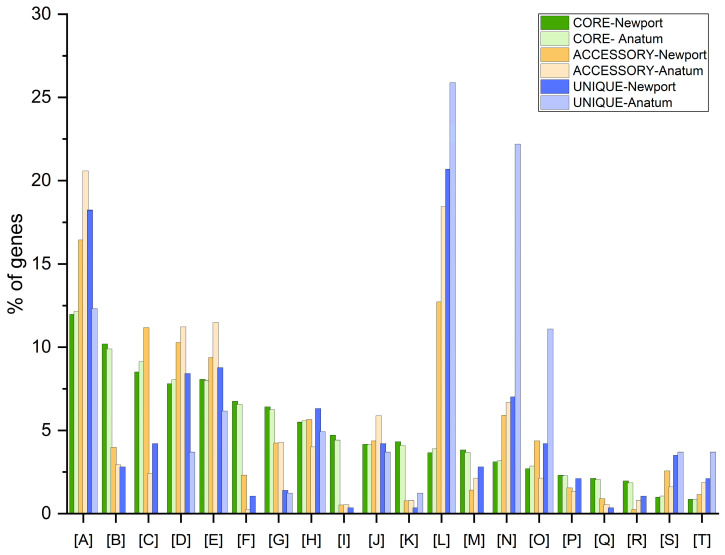
Distribution of the core, accessory, and unique genes of *Salmonella enterica* serotype Newport and Anatum according to gene functions. [A], general function prediction only; [B], amino acid transport and metabolism; [C], carbohydrate transport and metabolism; [D], transcription; [E], function unknown; [F], energy production and conversion; [G], inorganic ion transport and metabolism; [H], cell wall/membrane/envelope biogenesis; [I], translation, ribosomal structure and biogenesis; [J], signal transduction mechanisms; [K], coenzyme transport and metabolism; [L], replication, recombination and repair; [M], post-translational modification, and protein turnover and chaperones; [N], intracellular trafficking, secretion and vesicular transport; [O], cell motility; [P], lipid transport and metabolism; [Q], nucleotide transport and metabolism; [R], secondary metabolites biosynthesis, transport and catabolism; [S], defense mechanisms; [T], cell cycle control, cell division, and chromosome partitioning.

Figure 7 illustrates no significant differences in the relationship between serotypes for the CORE gene category. The highest percentages within this category were found in the following functions: general function prediction only ([A]), amino acid transport and metabolism ([B]), carbohydrate transport and metabolism ([C]), transcription ([D]), and, interestingly, function unknown ([E]). In the ACCESSORY gene category, serotype Anatum showed higher percentages in [A] and [E]; signal transduction mechanisms ([J]); and replication, recombination, and repair ([L]). In contrast, serotype Newport exhibited higher values in [B], [C], energy production and conversion ([F]), cell wall/membrane/envelope biogenesis ([H]), and cell motility ([O]). For the UNIQUE gene category, serotype Newport displayed a notable proportion of genes in [A], [D], [E], and [H]. Additionally, UNIQUE genes to Newport also appeared but not in Anatum are [B], [C], [F], post-translational modification, protein turnover and chaperones ([M]), and lipid transport and metabolism ([P]). Meanwhile, serotype Anatum had the highest proportions in replication, recombination, and repair ([L]); intracellular trafficking, secretion, and vesicular transport ([N]); and cell motility ([O]).

## 4. Discussion

Our results reveal a high prevalence of non-typhoidal *Salmonella enterica* strains in ground beef and pork from 20 of 28 Mexican states, with the Newport and Anatum serotypes being particularly notable. Of note, these states cover South, North, and Central Mexico. A total of 59 Newport strains (14.7%) and 50 Anatum strains (12.4%) were isolated from meat samples, aligning with previous reports showing that *Salmonella* serotypes are frequently associated with meat products, especially in urban areas with interconnected food supply chains [9,54,55,56].

In Mexico, the prevalence of *Salmonella* Anatum strains reported in this study aligns with or surpasses previous reports, such as the 8.9% found in ground beef from the state of Jalisco [57]. This highlights the persistent presence and increasing incidence of these serotypes in the country. The concentration of isolates in Mexico City (19 Newport and 15 Anatum isolates) points to a potential contamination nexus that requires further investigation. Urban areas are often hotspots for the spread of foodborne pathogens due to heightened consumer exposure and complex supply chains [58,59]. Other regional studies have reported similar proportions of these serotypes in different meat types, with Anatum at 9.4% in ground beef and 6.3% in ground pork, as well as Newport at 4.3% in ground beef and 7.2% in ground pork [24,28,60]. Several outbreaks of public health and animal food safety significance have been reported globally for Anatum [61,62,63] and Newport [64,65,66,67].

Comparative genomic analyses of the isolates revealed minimal genetic variation among Newport strains, with only three samples from the southern regions of Mexico (Tuxtla Gutierrez, Villahermosa, and Merida) showing a 1% variation compared to other Newport isolates. Anatum isolates demonstrated even lower genetic diversity, with less than 0.1% variation, suggesting a possible clonal spread of these strains within specific geographic areas. However, it is observed that the Newport strains are more widely distributed than the Anatum strains, the latter being mostly found in the central part of the country. This observation is consistent with the results of other studies highlighting the clonal nature of *Salmonella* populations and their adaptation to specific environments [8,68,69,70]. The strong correlation between the genomes of the isolates and their geographic origin highlights the localized transmission dynamics of *Salmonella* in Mexico. This suggests that specific public health interventions may be more effective if tailored to the unique characteristics of each region and serotype [71,72,73]. This localized approach emphasizes the importance of region-specific surveillance; it may improve the effectiveness of interventions to reduce infection rates, prevent outbreaks, and effectively control the spread of these pathogens.

A significant finding was the high prevalence of MDR strains, which accounted for 69% of those analyzed. This was particularly pronounced in the Newport serotype, where 87% of the strains were multidrug resistant. While the occurrence of resistance to clinically relevant antimicrobial agents was low—such as with ceftriaxone showing minimal resistance and ciprofloxacin showing none—it is crucial to note the high rates of resistance observed for tetracycline (68%), chloramphenicol (66%), sulfamethoxazole/trimethoprim (64%), and ampicillin (53%). Most of the MDR strains exhibited resistance to these antimicrobial agents. The strong association between *Salmonella* and these four agents underscores the potential for co-selection of resistance traits. Previous studies suggest that exposure to antimicrobial agents can enhance the survival of these microorganisms in both clinical and environmental settings [74,75,76,77]. Regarding resistance to azithromycin among *Salmonella* strains, 82% of serotype Newport demonstrated resistance to this macrolide antibiotic. Notably, three strains of serotype Anatum exhibited phenotypic resistance to azithromycin in different regions of Mexico, coinciding with the presence of serotype Newport in the same sample. These samples included two from ground beef in Tepic and Monterrey and one from ground pork in Mexico City. This observation supports the theory that *Salmonella* Newport could transfer resistance traits to *Salmonella* Anatum through mobile genetic elements [69]. A study observed that both serotypes showed phenotypic resistance in retail meat samples from the US [67]. This represents a significant public health concern, as azithromycin is a key antimicrobial agent for treating invasive non-typhoidal *Salmonella* (iNTS) infections [78,79].

Genomic analysis for serotyping using MLST has proven to be an effective tool for revealing genetic relationships and complex evolutionary groupings, complementing traditional serotyping methods [41]. The results of MLST are consistent with other studies conducted in Mexico, where ST64 was among the most prevalent, but not with ST118 [21,80,81,82]. These sequence types have been identified globally, with ST132 indicating a solid association with macrolide resistance [67,83,84,85,86,87,88]. This information can be verified using the EnteroBase database (https://enterobase.warwick.ac.uk/species/senterica/search_strains; accessed on 28 November 2024), which provides corroborative data from Mexico and globally.

In our analysis of genotypic resistance, we identified genes associated with resistance to 12 different groups of antimicrobial agents. Specifically, the *mdtB* and *mdtC* genes, which confer resistance to aminocoumarin, operate by promoting the expulsion of the drug via the expression of efflux pump proteins [89,90]. These genes were present in all strains of both serotypes, showing an identity percentage of 82% for both allelic variants. They have been reported in a wide range of Enterobacteria [91], although their origin appears to stem from a single gene duplication event in proteobacterial species [92]. While some publications have noted their presence in *Salmonella enterica*, they have not been found in the Anatum or Newport serotypes [93,94]. The aminoglycoside group exhibited the highest prevalence of resistance genes. The biochemical mechanism of this resistance involves modifying enzymes that inactivate these antibiotics, including acetyltransferases (AACs), adenyltransferases (AADs), and phosphotransferases (APHs) [24,95]. The *aac(6′)-ly* gene was detected in all sampled strains, while the *aadA2* gene was found in 49% of samples, indicating a significant level of resistance to aminoglycosides. This level is comparable to findings from other regional and worldwide studies related to foods of animal origin [24,76]. Additionally, other genes related to resistance against aminoglycosides and aminocoumarins, specifically the efflux pump genes *BaeR* and *CpxA*, were present in 100% of the strains analyzed [96,97]. Lastly, the *kdpE* gene, known to regulate virulence and enhance intracellular survival, was also found in all analyzed samples [98]. Although many strains were resistant to streptomycin, all strains of both serotypes were susceptible to amikacin, resulting in the effective use of this antimicrobial agent, even though they contained specific genes for this pharmacological group.

In the beta-lactam group, we identified five genetic determinants of resistance. The *ampH* gene, initially found in *E. coli* and associated with *ampC*, encodes a beta-lactam binding protein [99]. Among the 35 strains that carried this gene, only 8 exhibited resistance to AMP or AMC, indicating no causal relationship between the presence of this gene and genotypic resistance. The other four genes conferring beta-lactam resistance and producing class A beta-lactamases identified in this investigation—CARB-3, TEM-1, TEM-150, and SHV-134—have been widely reported in NTS strains in beef and pork [100,101]. Among the 29 strains carrying CARB-3, 4 with TEM-150, 3 with TEM-1, and 2 with SHV-134 presented resistance to AMP. Notably, these four genes were never found in the same strain, suggesting a distinct relationship between each gene and ampicillin resistance. Additionally, these microorganisms are widely distributed across all regions of the country. The CMY-59 gene, a type of beta-lactam resistance that inhibits cell wall synthesis, was initially found in *Shigella* and variants of *E. coli* and *Klebsiella* [102,103]. It has recently been detected in retail meat from NTS strains [104]. Notably, one strain from Newport traced back to a sample of ground beef in Toluca carried this gene. This strain is the only ST45 strain identified and does not have other beta-lactam-resistance genes. However, it does demonstrate phenotypic resistance to ampicillin, amoxicillin/clavulanic acid, and, interesting, ceftriaxone, suggesting that this resistance is likely due to the presence of the CMY-59 gene but not to cefepime, which shows susceptibility, not only in this strain but in all microorganisms analyzed.

Quinolones and fluoroquinolones are two groups of widely described antimicrobial agents that inhibit DNA topoisomerase VI and DNA gyrase synthesis but currently exhibit resistance to these drugs via the mutation of chromosomal *gyr* and *par* genes of the quinolone resistance-determining regions (QRDRs) and by acquisition of plasmid-mediated quinolone resistance (PMQR) *qnr* genes protecting quinolone enzymes [76,105]. There are other mechanisms for fluoroquinolone efflux via transport to the outside of the membrane in *Salmonella*; some of the genes encoding for this mechanism are *mdtK* and the *emr* genes family [106,107]. These last genes that are related to multidrug resistance by efflux pumps were present in 100% of the samples, and the three related to PMQR (*qnrA1*, *qnrB5*, and *qnrB20*) present in 65% of the isolates cannot be associated with phenotypic resistance because all samples were susceptible to ciprofloxacin. In Mexico, good susceptibility to fluoroquinolones has been reported with few resistant *Salmonella* isolates in animal food [20,24].

In North America, macrolide resistance is of great interest due to outbreaks of *Salmonella* Anatum in various food products, mainly of animal origin, such as beef [23,24]. Although the World Health Organization (WHO) and the World Organisation for Animal Health (OIE) have pushed for limitations on its veterinary use by including it as critically important, it is not yet available for veterinary use [108] and, in Europe and the USA, have taken restrictive measures for its therapeutic use in farm animals for human consumption since its predominant use is for the treatment of bacterial infections when the patient is allergic to penicillin [109,110,111]. In Mexico, several macrolides are approved for veterinary use (josamycin, erythromycin, troleandomycin, tylosin, oleandomycin, tilmicosin, and rosaramicin) with medium importance, and they can be supplied with a simple prescription for veterinary use [112]. Several resistance mechanisms have been reported for macrolides, such as 23S rRNA mutation, methylation, efflux pumps, peptidyl tRNA hydrolase overexpression, and enzymatic modification of the macrolide. One of the most important is related to the *mph* genes coding for the resistance enzyme MPH(2′)-I that inactivates macrolides, preferably 14- and 16-membered, by phosphorylation [113,114,115]. The present study found a strong association between the *mphA* gene, present only in the Newport serotype, and resistance to azithromycin, as the 35 strains in which this gene was present were all resistant to this antibiotic. This finding is in agreement with what has been reported by our research group in beef previously [24] and in other regions of the world with different animal and environmental food matrices, mainly in *Salmonella* Newport [8,67,106,116,117,118].

We found that phenicols are one of the groups of antimicrobials with the highest prevalence of resistance in Mexico in ground beef and pork, being present in 66% of the isolates of *Salmonella* Newport and Anatum. This result is congruent with previous reports for Mexico [24,57,119]. One of the main resistance mechanisms used by bacterial cells to survive phenicols is reducing the intracellular concentration by expelling the substances via efflux pumps; the genes encoding this mechanism are *floR* and *cmlA* [120,121]. We found 48 *Salmonella* strains with the presence of *floR*; all of these strains were resistant to chloramphenicol, indicating a clear association.

The sulfonamide group is likely to be one of the antibiotics with the highest reported resistance rates, given that it was one of the first to be marketed. Currently, it is combined with trimethoprim to inhibit the synthesis of folate, essential for bacterial growth. In the case of sulfonamides, it has been reported that the *sul* family of genes encoding dihydropteroate synthase (DHPS) mutant enzymes can prevent sulfonamide binding at the active site [122]. In the case of trimethoprim-resistant dihydrofolate reductase (DHFR), enzymes are expressed by the *dfrA* gene family [123]. These two groups of genes were found to be distributed in 82% of Newport and 56% in Anatum; of note, in the 44 strains where *sul1* was observed, there was 100% resistance to sulfamethoxazole. On the other hand, of the 17 strains with the presence of *sul2*, 6 isolates coincided with both genes, and in the remaining 11, there was resistance in only 2 isolates (both serotype Anatum). Fortunately, the remaining nine strains were susceptible, indicating that the resistance to this antibiotic is due to the *sul1* gene. Regarding the *dfrA* gene family, it was observed that only the *dfrA12* and *dfrA17* genes were present in Anatum, and *dfrA1* and *dfrA19* in Anatum. This finding suggests that these genes have not been disseminated among the different subspecies; in the nine strains of *Salmonella* Anatum where the *dfrA12* (8 strains) and *dfrA19* (1 strain) genes were present, there was resistance to sulfamethoxazole/trimethoprim in the central region of Mexico (Mexico City, Puebla, Tlaxcala, Toluca, and Chilpancingo). A similar result was observed with *Salmonella* Anatum, wherein, regarding the 34 strains where *dfrA1* was present, all were resistant. Still, in this case, this serotype was found dispersed in all regions of Mexico. These findings align with those reported in other works in the region related to *Salmonella*; however, there are few reports concerning Anatum [57,119].

Resistance to tetracycline (one of the broad-spectrum antimicrobials) has been observed in *Salmonella enterica* from food of animal origin [77,104,124]. Several types of *tet* family genes of classes A, B, C, D, and G have been reported in the three mechanisms of tetracycline resistance, efflux pumps, ribosomal protection, and enzymatic inactivation [125]. The incidence rate in Mexican meat is high (~40%) [19,23,53,115]; we determined the presence of three *tet* genes: *tetA*, *tetB*, and *tetD*. The *tetA* gene was the most abundant, with 62% (48 isolates), and there was a strong association with tetracycline resistance since only two strains (4%) were susceptible to this drug. Interestingly, the same pattern of presence or absence was observed for *sul1* and *dfrA* genes, so there are strong indications that, in these strains, there is the same mechanism of expression of intrinsic resistance [125]. Only two strains of *Salmonella* Anatum, in Toluca and Tlaxcala, presented the *tetB* gene, again drawing attention to the fact that these strains, which had phenotypic resistance, are growing in concern in the central region of Mexico. Finally, for the *tetD* gene, two strains of *Salmonella* Newport were found in the city of Durango, both resistant to tetracycline, from ground beef and ground pork, thus reinforcing the theory of poor hygienic conditions at the site due to cross-contamination of the samples.

Pangenome analysis can be useful to understand adaptations to the environment at the structural, functional, and genetic level of *Salmonella enterica* serotypes Newport and Anatum, influencing their survivability, invasiveness, and pathogenicity. It has been suggested that modifying or varying the pangenome in specific proportions may manifest in species-specific or even serotype-specific adaptations in the expression of specialized metabolism to resist different environmental conditions, virulence factors, and antimicrobial resistance itself [126,127,128]. The genes present in the CORE genome of both serotypes were predominantly for essential functions of maintaining basic cellular and survival processes, such as amino acid and carbohydrate transport and metabolism, as well as transcription. These results of the conserved nature of the genes agree with other pangenome studies in *Salmonella* [129,130,131]. Amino acid metabolism may influence virulence and the ability to colonize in different environments [132]. This may suggest the ability of *Salmonella* Newport to have more remarkable persistence in resource use and metabolic efficiency due to the slight differences found with Anatum. Notably, a large proportion of genes in both serotypes belong to domains of unknown function (DUFs), suggesting the possibility that lack of characterization is associated with specialized pathogenicity and stressor-resistance functions [133,134].

The functions of membrane biogenesis and carbohydrate transport and metabolism were overrepresented in the ACCESSORY genes in *Salmonella* Newport, which could suggest a better adaptation to changing conditions since membrane biogenesis is a fundamental factor for adaptation to changes in temperature and improving its resistance [135].

The Newport serotype has a broader distribution, with more isolates in both beef and pork and more antibiotic-resistance genes than the Anatum serotype. As for UNIQUE genes, *Salmonella* Anatum showed a notorious proportion of functions related to cell motility and intracellular trafficking, which, due to its vast presence in ground beef, shows greater tissue colonization. On the other hand, *Salmonella* Newport presents UNIQUE genes associated with energy production and conversion, as well as lipid transport, giving it a remarkable ability to survive in environments with limited resources and environmental pressures by adjusting its energy production to lipid transport [131,136].

Finally, the evaluation of the pangenome of the two *Salmonella* serotypes suggests a slight divergent evolution favored by transmission, in this case, the type of meat and its ability to adapt. An in-depth investigation of unknown functions is essential, as these could be key to understanding virulence factors and the ability to resist antimicrobial stresses in food production systems.

## 5. Conclusions

In conclusion, we observed that these two serotypes are present in beef and pork sold to the population in Mexico. However, *Salmonella* Newport has a broader distribution than Anatum. This capacity of Newport to distribute itself may be related to its greater capacity to survive in adverse environments compared to Anatum, in addition to presenting a better capacity of resistance to antibiotics, likely due to the presence of more antibiotic-resistance genes. For instance, *mphA*, which confers resistance to macrolides, and the *ramA* gene, which confers the capacity for multidrug resistance, were not found in the Anatum serotype. The findings presented here highlight the risk of these two serotypes to public and environmental health and underscore the need for robust surveillance programs that provide information for developing stricter public policies. It is important to remark that the presence of these serotypes affects both animal and human health and, consequently, the local economy. Finally, other environments where these serotypes could be present should be studied, as well as their possible effects related to antimicrobial resistance.

## Data Availability

The original SRA sequences presented in the study are available in the National Center for Biotechnology Information at https://www.ncbi.nlm.nih.gov/ (accessed on 28 November 2024) under the BioProyect Accession PRJNA480281; Table S2 shows the Run Accession and BioSample of the samples in NCBI.

## Supplementary Materials

The following supporting information can be downloaded at https://www.mdpi.com/article/10.3390/microorganisms12122485/s1, Figure S1: Number of *Salmonella* Newport and Anatum isolates in the main cities of Mexico from ground beef and pork; Table S1: Cities and states where ground beef and pork samples were collected in Mexico; Table S2: Relationship of beef and pork samples (ID) concerning their Run Accession and BioSample code deposited at NCBI.
