# Supplementary material for: Geography, Antimicrobial Resistance, and Genomics of Salmonella enterica (Serotypes Newport and Anatum) from Meat in Mexico (2021–2023)
Source: Microorganisms. 2024 Dec 3;12(12):2485. doi: 10.3390/microorganisms12122485 (PMC11727726; doi:10.3390/microorganisms12122485)
Supplement: Supplementary file 1 [file microorganisms-12-02485-s001.zip › microorganisms-3340132-supplementary.pdf]

microorganisms

MDPI

Supplementary Materials

Geography, Antimicrobial Resistance, and Genomics of *Salmonella enterica* (Serotypes Newport and Anatum) from Meat in Mexico (2021–2023)

Eduardo Canek Reynoso [1], Enrique Jesús Delgado-Suárez [2], Cindy Fabiola Hernández-Pérez [3], Yaselda Chavarin-Pineda [4], Elizabeth Ernestina Godoy-Lozano [5], Geny Fierros-Zárate [1], Omar Alejandro Aguilar-Vera [6], Santiago Castillo-Ramírez [6], Luz del Carmen Sierra Gómez-Pedroso [2] and Luisa María Sánchez-Zamorano [1,*]

[1] Centro de Investigación en Salud Poblacional, Instituto Nacional de Salud Pública (INSP), Morelos 62100, Mexico; eduardo.reynoso@insp.edu.mx (E.C.R.); gsfierro@insp.mx (G.F.-Z.)
[2] Facultad de Medicina Veterinaria y Zootecnia, Universidad Nacional Autónoma de México (UNAM), Ciudad de México 04510, Mexico; ejds@fmvz.unam.mx (E.J.D.-S.); luzsierra@fmvz.unam.mx (L.d.C.S.G.-P.)
[3] Centro Nacional de Referencia de Inocuidad y Bioseguridad Agroalimentaria, Servicio Nacional de Sanidad, Inocuidad y Calidad Agroalimentaria (SENASICA), Estado de México 55740, Mexico; dgiaap.iica44@senasica.gob.mx
[4] Centro de Investigación en Ciencias Agrícolas, Instituto de Ciencias, Benemérita Universidad Autónoma de Puebla (BUAP), Puebla 72570, Mexico
[5] Centro de Investigación Sobre Enfermedades Infecciosas, Instituto Nacional de Salud Pública (INSP), Morelos 62100, Mexico; elizabeth.godoy@insp.mx
[6] Centro de Ciencias Genómicas, Universidad Nacional Autónoma de México (UNAM), Morelos 62210, Mexico; aaguilar@ccg.unam.mx (O.A.A.-V.); iago@ccg.unam.mx (S.C.-R.)
* Correspondence: szamoran@insp.mx; Tel.: +52-777-101-29-79

Table S1. Cities and states where ground beef and pork samples were collected in Mexico.

City	State	Average temperature (°C)	Season
Ciudad de Mexico	Ciudad de Mexico	17.8	Spring 2021
Cuernavaca	Morelos	21.8	Spring 2021
Puebla	Puebla	20.0	Spring 2021
Culiacan	Sinaloa	19.1	Spring 2023
Tepic	Nayarit	19.9	Spring 2023
Aguascalientes	Aguascalientes	17.8	Spring 2023
Guanajuato	Guanajuato	22.5	Spring 2023
Monterrey	Nuevo Leon	27.4	Summer 2022
Queretaro	Queretaro	17.5	Summer 2021
Pachuca	Hidalgo	15.5	Summer 2021
Toluca	Estado de Mexico	15.4	Summer 2021
Tlaxcala	Tlaxcala	16.0	Summer 2021
Chilpancingo	Guerrero	17.6	Summer 2021
Campeche	Campeche	27.2	Summer 2023
Chetumal	Quintana Roo	31.7	Summer 2023
Merida	Yucatan	25.2	Autumn 2021
Tuxtla Gutierrez	Chiapas	25.8	Autumn 2022
Zacatecas	Zacatecas	20.1	Autumn 2022
Mexicali	Baja California	21.0	Autumn 2022
Durango	Durango	16.8	Autumn 2022
Oaxaca	Oaxaca	17.0	Autumn 2022
Villahermosa	Tabasco	28.6	Autumn 2022

Hermosillo	Sonora	21.6	Winter 2022
Torreon	Coahuila	24.1	Winter 2022
Puerto Veracruz	Veracruz	24.9	Winter 2022
La Paz	Baja California Sur	22.5	Winter 2023
Tampico	Tamaulipas	18.4	Winter 2023
Morelia	Michoacán	13.9	Winter 2023

Table S2. Relationship of beef and pork samples (ID) concerning their Run Accession and BioSample code deposited at NCBI.

Sample ID	Run	BioSample
GB1-1-CdMx21	SRR18856396	SAMN27723560
GB12-1-Pue21	SRR18856402	SAMN27723590
GB12-2-Pue21	SRR18856429	SAMN27723557
GB24-1-CdMx21	SRR20081756	SAMN29633025
GB25-2-CdMx21	SRR23930570	SAMN33796094
GB27-1-CdMx21	SRR20081779	SAMN29633022
GB29-3-Mex21	SRR20081776	SAMN29633033
GB31-1-Mex21	SRR20081769	SAMN29633040
GB31-3-Mex21	SRR20081766	SAMN29633042
GB33-1-Mex21	SRR20081759	SAMN29633049
GB35-1-Mex21	SRR20081754	SAMN29633053
GB35-2-Mex21	SRR20081753	SAMN29633054
GB37-2-Mex21	SRR20081749	SAMN29633058
GB38-1-Mex21	SRR20081748	SAMN29633059
GB39-1-Mex21	SRR20081743	SAMN29633063
GB42-1-Tlax21	SRR20081732	SAMN29633073
GB43-1-Qro21	SRR20081731	SAMN29633074
GB45-1-Gro21	SRR20081730	SAMN29633075
GB48-1-Gro21	SRR20081728	SAMN29633077
GB49-1-Gro21	SRR20081727	SAMN29633078
GB5-2-Mor21	SRR18856425	SAMN27723569
GB56-2-Hgo21	SRR20081713	SAMN29633091
GB57-1-Chia21	SRR20081710	SAMN29633093
GB6-2-Mor21	SRR18856414	SAMN27723579
GB63-3-Yuc21	SRR26379211	SAMN37792023
GB65-1-Yuc21	SRR26379204	SAMN37792027
GB71-1-NLe22	SRR26379157	SAMN37792070
GB72-1-NLe22	SRR26379156	SAMN37792071
GB7-3-Mor21	SRR18856418	SAMN27723558
GB73-1-NLe22	SRR26379154	SAMN37792073
GB73-2-NLe22	SRR26379153	SAMN37792074
GB75-1-Tab22	SRR26379143	SAMN37792083
GB86-1-Chia22	SRR26379261	SAMN37792134
GB91-1-Dgo22	SRR26379219	SAMN37792159
GB92-2-Dgo22	SRR26379218	SAMN37792160
GB96-1-Son22	SRR29467008	SAMN41896422
GB102-1-Ags23	SRR29466981	SAMN41896440
GB103-1-Ags23	SRR29466977	SAMN41896443
GB103-2-Ags23	SRR29466976	SAMN41896444
GB103-3-Ags23	SRR29466975	SAMN41896445
GB105-2-Ags23	SRR29466972	SAMN41896448
GB107-1-Nay23	SRR29466955	SAMN41896463
GB107-2-Nay23	SRR29466954	SAMN41896464
GB109-2-Nay23	SRR29466950	SAMN41896468
GB110-1-Nay23	SRR29466949	SAMN41896469
GB119-2-Camp23	SRR29466994	SAMN41896500
GP10-1-Mor22	SRR26379177	SAMN37792052

GP10-2-Mor22	SRR26379176	SAMN37792053
GP10-3-Mor22	SRR26379175	SAMN37792054
GP10-4-Mor22	SRR26379174	SAMN37792055
GP12-1-Mor22	SRR26379171	SAMN37792057
GP12-2-Mor22	SRR26379170	SAMN37792058
GP18-1-Mor22	SRR26379164	SAMN37792064
GP20-3-Mor21	SRR26379159	SAMN37792068
GP21-1-Mor21	SRR26379158	SAMN37792069
GP22-1-NLe22	SRR26379152	SAMN37792075
GP23-1-NLe22	SRR26379149	SAMN37792077
GP34-1-Oax22	SRR26379249	SAMN37792097
GP48-1-CdMx22	SRR26379240	SAMN37792141
GP48-2-CdMx22	SRR26379238	SAMN37792142
GP49-1-CdMx22	SRR26379237	SAMN37792143
GP52-1-CdMx22	SRR26379230	SAMN37792149
GP53-1-CdMx22	SRR26379227	SAMN37792152
GP55-1-Dgo22	SRR26379220	SAMN37792158
GP6-1-Ver22	SRR26379191	SAMN37792039
GP8-2-Mor22	SRR26379180	SAMN37792049
GP60-1-Son22	SRR29466923	SAMN41896412
GP72-1-Ags23	SRR29466963	SAMN41896456
GP72-2-Ags23	SRR29466962	SAMN41896457
GP79-3-Gto23	SRR29466928	SAMN41896488
GP80-1-Gto23	SRR29466927	SAMN41896489
GP80-2-Gto23	SRR29466926	SAMN41896490
GP81-1-Gto23	SRR29466925	SAMN41896491
GP81-2-Gto23	SRR29466924	SAMN41896492
GP82-1-Gto23	SRR29466922	SAMN41896493
GP83-1-Gto23	SRR29466921	SAMN41896494
GP88-1-Camp23	SRR29467027	SAMN41896507
GB63-3-Yuc21	SRR18856396	SAMN27723560
GB65-1-Yuc21	SRR18856402	SAMN27723590
GB71-1-NLe22	SRR18856429	SAMN27723557
GB72-1-NLe22	SRR20081756	SAMN29633025
GB7-3-Mor21	SRR23930570	SAMN33796094
GB73-1-NLe22	SRR20081779	SAMN29633022
GB73-2-NLe22	SRR20081776	SAMN29633033
GB75-1-Tab22	SRR20081769	SAMN29633040
GB86-1-Chia22	SRR20081766	SAMN29633042
GB91-1-Dgo22	SRR20081759	SAMN29633049
GB92-2-Dgo22	SRR20081754	SAMN29633053

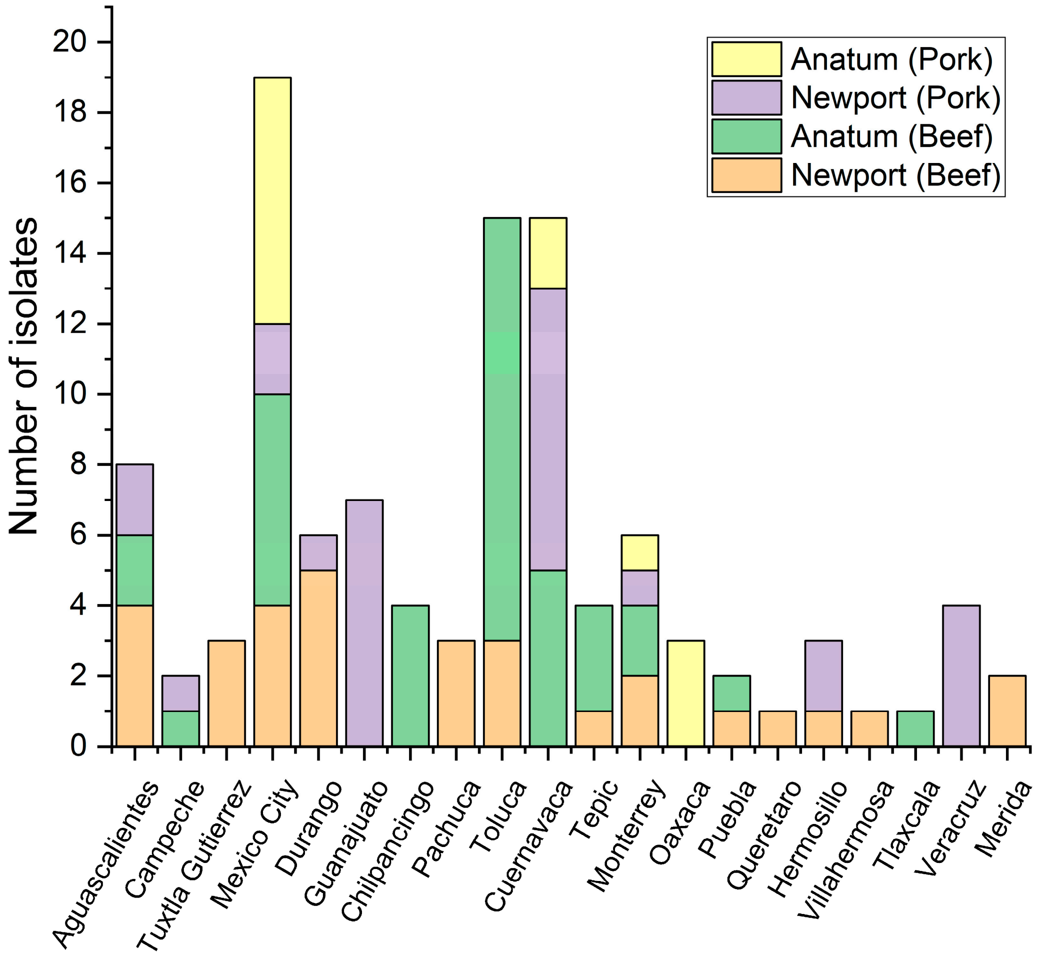

Figure S1. Number of *Salmonella* Newport and Anatum isolates in the main cities of Mexico from ground beef and pork.
